# Utility of Percutaneous Needle Tenotomy to Reduce Pain and Improve Function in Common Extensor Tendinosis of the Lateral Epicondyle

**DOI:** 10.31486/toj.21.0044

**Published:** 2021

**Authors:** Nicolas S. Hatamiya, Yuka Kobayashi, Andrew W. Gottschalk

**Affiliations:** ^1^ Department of Family Medicine, Division of Sports Medicine, University of California–Los Angeles, Los Angeles, CA; ^2^ Department of Family and Sports Medicine, Oregon Health and Sciences University, Portland, OR; ^3^ Department of Orthopedics, Sports Medicine Institute, Ochsner Clinic Foundation, New Orleans, LA

## CASE PRESENTATION

A 20-year-old, right-hand-dominant female collegiate tennis player presents with persistent pain over her right lateral epicondyle for the past 4 months that began when she increased activity at the start of season. She was initially diagnosed with lateral epicondylitis and tried oral anti-inflammatories, physical therapy, a counterforce brace, a corticosteroid injection, and activity modification without relief. Point-of-care ultrasound demonstrated evidence of chronic tendinosis with thickening of the common extensor tendon, focal hypoechogenicity, and neovascularization. She did a trial of topical nitroglycerin patches and eccentric exercises without significant improvement. She prefers to avoid surgery and would like to know the efficacy of percutaneous needle tenotomy for her common extensor tendinosis.

## BACKGROUND

Pain at the lateral epicondyle is a common condition, affecting nearly 1% to 3% of adults each year.^1,2^ Acute pain at the lateral epicondyle is usually a self-limiting condition, but several interventions and treatment options have been explored to advance recovery and promote healing. For a systematic review published in 2011, Bisset et al reviewed 80 studies and concluded that topical nonsteroidal anti-inflammatory drugs (NSAIDs) and local corticosteroid injections are likely to be beneficial for short-term pain relief; low-level laser therapy is likely to be beneficial for short-term pain relief and improvement in function; but the efficacy of oral NSAIDs, acupuncture, combination physical therapy, orthosis, manipulation, iontophoresis, pulsed electromagnetic field treatment, and exercise is unknown.^3^

Cases of pain at the lateral epicondyle that do not respond to conservative measures are challenging to treat, with 1 in 10 patients requiring surgical intervention.^4^ Chronically injured tendons may have histopathologic degenerative changes in tendon architecture, characterized by hypercellularity, increased vascularization, and disorganization of collagen.^5^ This chronic tendon state is commonly referred to as tendinosis or tendinopathy.^6,7^

In percutaneous needle tenotomy—an emerging nonsurgical treatment for chronic tendinosis—a needle is used to repetitively fenestrate a diseased tendon to disrupt scar tissue and induce bleeding. This intervention leads to an inflammatory state caused by activation of the clotting cascade and a release of growth factors, converting the chronic injury to an acute healing state.^8^ This technique has been used for chronic tendinosis in multiple anatomic locations, including the common extensor tendon at the lateral epicondyle.^9^

## REVIEW OF EVIDENCE

Altay et al first described the concept of repetitively inserting a needle percutaneously into an area of diseased tendon at the lateral epicondyle in 2002.^10^ They used a peppering injection technique in which a needle is inserted, withdrawn, redirected, and reinserted at the point of maximal tenderness along the lateral epicondyle 40 to 50 times, while “a sensation like crepitation or cracking is felt and the injections are continued until this sensation is lost.”^10^ Altay et al compared the peppering technique in 2 groups of patients diagnosed with lateral epicondylitis: one group received lidocaine only, and the second group received lidocaine plus triamcinolone. Compared to baseline, both groups exhibited excellent improvements in Verhaar scores, which subjectively measure pain, function, and patient satisfaction, and no statistically significant differences were found between the groups.^10^

In 2009, Dogramaci et al further investigated the use of the peppering technique for chronic pain at the lateral epicondyle in a prospective randomized study that compared 3 injection procedures: steroid with anesthetic, anesthetic plus peppering technique, and steroid with anesthetic plus peppering technique.^11^ All groups had reductions in pain, as measured via the visual analog scale (VAS) at 3-week and 6-month follow-ups, compared to their pretreatment condition. The steroid-anesthetic-peppering group had statistically significant improvements in Verhaar scores (84% excellent) compared to the steroid plus anesthetic without peppering (36% excellent; *P*=0.002) and the anesthetic alone with peppering (48% excellent; *P*=0.011) groups.^11^ The authors concluded that incorporating a peppering technique with steroid injections may be considered a first-choice treatment for lateral epicondyle pain.^11^

A systematic review published in 2017 by Mattie et al analyzed 6 studies that investigated the use of percutaneous needle tenotomy for the treatment of lateral epicondylitis: 1 prospective randomized controlled trial, 4 prospective studies, and 1 retrospective study.^12^ The studies included in the Mattie et al review had heterogeneity in their percutaneous needling procedures (Table). Five studies supported the efficacy of percutaneous needle tenotomy for chronic tendon pain at the lateral epicondyle,^13-17^ and one study by Barnes et al demonstrated significant improvements in pain (via VAS) and function (via the Mayo Elbow Performance score and the Quick Disabilities of the Arm, Shoulder and Hand [QuickDASH] score).^18^ Barnes et al concluded that they could not clearly recommend percutaneous needle tenotomy for the treatment of common extensor tendinosis of the lateral epicondyle because of the lack of randomized sham- or placebo-controlled trials. However, they noted that percutaneous needle tenotomy presents an alternative to surgical release of the common extensor tendinosis at the lateral epicondyle.

**Table.** Details of Tenotomy Procedures

Study	Procedure Description
McShane et al, 2006^13^	Ultrasound-guided percutaneous needle tenotomy was performed using an 18- or 20-gauge needle to repeatedly fenestrate the tendon with concomitant corticosteroid injection (6 mg betamethasone [Celestone Soluspan] or 40 mg triamcinolone acetonide). The tendon was treated until palpable softening of the tissue occurred and sonographic visualization showed that the entire tendon had been treated. The needle was then reinserted to abrade the periosteum of the lateral epicondyle.
Lakhey et al, 2007^14^	The common extensor tendon was infiltrated with 10 mL 0.2% ropivacaine using a 30-gauge needle, and then percutaneous needle tenotomy was performed by dividing the origin of the common extensor tendon at the site of maximal tenderness using the bevel of an 18-gauge needle.
McShane et al, 2008^15^	Subcutaneous tissues overlying the lateral epicondyle were infiltrated with 0.5% bupivacaine and 1% sodium bicarbonate using a 22-gauge needle, and then ultrasound-guided percutaneous needle tenotomy was performed using a 20-gauge needle to repeatedly fenestrate the tendon until palpable softening of the tissue occurred and sonographic visualization showed that the entire tendon had been treated. The needle was then reinserted to abrade the periosteum of the lateral epicondyle.
Zhu et al, 2008^16^	Percutaneous needle tenotomy was performed using a 16-gauge needle to repeatedly fenestrate the common extensor tendon with subsequent corticosteroid injection, a 1 mL mixture of 25 mg prednisone acetate and 1% lidocaine.
Stenhouse et al, 2013^17^	Percutaneous needle tenotomy alone was compared to percutaneous needle tenotomy with autologous conditioned plasma injection, for which whole blood was drawn and centrifuged once at 1,500 rpm for 5 minutes, and then the platelet-containing plasma layer was drawn and injected. Percutaneous tenotomy was performed using a 23-gauge needle to pepper the tendon 40 to 50 times.
Barnes et al, 2015^18^	Ultrasound-guided percutaneous needle tenotomy was performed with the TX1 system (Tenex Health Inc), a proprietary needle-like device that uses ultrasonic energy to rapidly oscillate an 18-gauge tip to debride diseased tissue and remove it through an inflow-outflow circuit. To anesthetize the subcutaneous and insertion region, 3 mL 1% lidocaine was used.

rpm, revolutions per minute.

Other studies have investigated the utility of different percutaneous needling techniques using platelet-rich plasma (PRP) injections for recalcitrant lateral epicondylitis. In 2017, Gaspar et al compared needle fenestration in which a needle was used to perforate the common extensor origin 5 times (n=45) vs percutaneous needle tenotomy (n=48) in which the beveled edge of a needle tip was used to abrade the footprint of the common extensor tendon.^19^ Both groups received concomitant PRP injection. At a mean follow-up of 40 months, both groups had significant improvements in VAS score (mean –6.1, 95% CI –6.8 to –5.5; *P*<0.0001); QuickDASH score (mean –46, 95% CI –52 to –40; *P*<0.0001); Patient-Rated Tennis Elbow Evaluation score (mean –57, 95% CI –64 to –50; *P*<0.0001); and grip strength (mean +6.1 kg, 95% CI 4.9 to 7.3; *P*<0.0001), with no significant differences noted between the fenestration and tenotomy groups.^19^ No complications were reported in either group. Interestingly, 22% in the fenestration group underwent subsequent procedures to treat recurrent symptoms compared to 10% in the tenotomy group (*P*=0.05), suggesting that percutaneous needle tenotomy was less likely to require conversion to open tenotomy compared to needle fenestration.^19^

In 2019, Martin et al investigated clinical improvements and tendon structure changes in chronic lateral epicondylopathy 20 months after percutaneous needle tenotomy with PRP (n=27) and tenotomy with lidocaine (n=24).^20^ Subjects in both groups showed meaningful improvements in VAS (90.91%) and Disabilities of the Arm, Shoulder and Hand (DASH-E) scores (80.85%), with no significant difference between groups.^20^ Tenotomy-induced changes in tendon structure occurred. Overall, significant decreases in tendon thickness (*P*=0.0006), vascularity (*P*<0.0001), and echotexture (*P*<0.0001) were noted from baseline.^20^ The study authors concluded that percutaneous tenotomy with PRP was not superior to percutaneous tenotomy with lidocaine in terms of reducing pain and disability at 20 months in patients with recalcitrant elbow epicondylopathy.^20^

## TAKEAWAY

High-quality studies investigating the use of percutaneous needle tenotomy for chronic tendinosis at the lateral epicondyle are limited. The existing literature suggests that percutaneous needle tenotomy may decrease pain and improve function for common extensor tendinosis at the lateral epicondyle for recalcitrant tendinopathy that has failed other conservative measures. However, further prospective, randomized controlled trials are warranted. Percutaneous needle tenotomy remains a relatively safe and minimally invasive procedure that may serve as an alternative treatment option prior to considering surgical release.

## CASE RESOLUTION

The athlete had an ultrasound-guided percutaneous needle tenotomy performed in clinic, targeted toward the common extensor tendons of the lateral epicondyle. We injected 3 mL 1% lidocaine without epinephrine into the tendon sheath and used a 22-gauge, 1.5-inch needle to make 40 passes using the peppering technique. At 6-month follow-up, she reported a reduction in pain and an improvement in function. She was able to successfully return to competition the following season and avoid surgery.

## Notes

*This article meets the Accreditation Council for Graduate Medical Education and the American Board of Medical Specialties Maintenance of Certification competencies for Patient Care and Medical Knowledge.*
